# Resting Energy Expenditure in Adults with Becker’s Muscular Dystrophy

**DOI:** 10.1371/journal.pone.0169848

**Published:** 2017-01-06

**Authors:** Matthew F. Jacques, Paul Orme, Jonathon Smith, Christopher I. Morse

**Affiliations:** 1 Health, Exercise & Active Living (HEAL) Research Centre, Manchester Metropolitan University, Cheshire Campus, Crewe, United Kingdom; 2 The Neuromuscular Centre, Winsford, Cheshire, United Kingdom; Rutgers University Newark, UNITED STATES

## Abstract

**Purpose:**

The purpose of this study was: 1) To compare Resting energy expenditure (REE) in adult males with Becker’s Muscular Dystrophy (BeMD, n = 21, 39 ±12 years) and healthy controls (CTRL, n = 12, 37 ±12 years) 2) Determine whether other physiological parameters correlate with REE in BeMD, and 3) Compare current prediction methods of REE with measured REE.

**Methods:**

REE was calculated via indirect calorimetry using continuous, expired gas analysis following an overnight fast. Fat free mass (FFM) and fat mass were measured by bioelectrical impedance. B-mode ultrasound measured Tibialis Anterior (TA) and Gastrocnemius Medialis (GM) anatomical cross sectional area (ACSA). The Bone Specific Physical Activity Questionnaire measured physical activity.

**Results:**

No difference in REE was found between CTRL and BeMD groups (1913 ±203 & 1786 ±324 Kcal respectively). Other physiological comparisons showed increased fat mass (+54%), decreased TA ACSA (-42%), increased GM ACSA (+25%) as well as reduced respiratory function (FVC -28%; FEV1−27%) in BeMD adults compared to controls. REE estimated from prediction equations (Schofield’s) in Muscular Dystrophy were different from measured REE (P<0.05, bias = -728kcal), while the Mifflin equation was no different from measured REE (r^2^ = 0.58, Bias = -8kcal). Within the present BeMD, REE predicted from FFM (REE = FFM x 34.57–270; r^2^ = 0.85) and body mass (REE = BM x 15.65 + 421.5; r^2^ = 0.66), were not different from measured REE (bias equals 0 and 0.2kcals, respectively)

**Conclusions:**

Despite no differences in REE between CTRL and BeMD adults, increased fat masses highlights the requirement for explicit nutritional guidelines, as well as maintenance of physical activity levels, where possible. Prediction equations are frequently used in clinical settings, however these have been shown to be less accurate in BeMD; therefore, the equations proposed here should be used where possible.

## 1. Introduction

Muscular dystrophy (MD) is a broad group of myogenic recessive muscle disorders, with variable severity [1]. Duchenne (DMD) and Beckers (BeMD) Muscular Dystrophy are characterised by the absence or reduced expression of the cytoskeletal protein dystrophin, resulting in progressive muscle degeneration[2]. Duchenne Muscular Dystrophy (DMD), characterised by non-functioning dystrophin, is the most severe form of MD, with an estimated incidence of 3 in 100,000 boys [3]. Becker’s muscular Dystrophy (BeMD) evidences partially functioning dystrophin, and is therefore a milder yet more variable form of dystrophinopathy, with an incidence of 2 in 100,000 male births [3]. Shimizu-Fujiwara et al [4] estimated that children with DMD had decreasing physical activity levels, resulting in reduced calorific energy requirements, therefore reducing their daily calorific intake needs. In contrast, the variable nature and relative degree of physical disability within the BeMD population may be less suitable to a “one size fits all” approach to daily calorific guidelines.

Nutritional support and knowledge of Resting energy expenditure (REE) is essential for individuals with limited physical activity in order to provide guidance on calorific intake and to limit excessive weight gain [5]. However, unlike DMD, there are no current studies of REE or its estimation from anthropometric measures such as body mass or fat free mass in adult males with BeMD. REE, here defined as the energy used in maintaining bodily functions such as respiration, circulation, cellular metabolism and body temperature in a fasted, thermo-neutral state [6], is best predicted in healthy population by FFM, with correlations ranging between 65–90% [7, 8]. In addition, REE can be normalised to FFM to reduce the impact of body size on REE [9].

In DMD, the loss of FFM is attributed with a lower REE than non-dystrophic controls [10, 11], with the progressive nature of DMD, resulting in the loss of FFM, leads to REE to decrease with age within DMD [4]. It is likely therefore, that REE will be lower in adults with BeMD, who also experience a loss of muscle mass. Loss of muscle mass is a consequence of the progression of MDs, however lower physical activity levels associated with forms of MD may also lead to increased muscle atrophy [12]. However, there is currently no research available in either muscle mass or REE in people with BeMD of any age. REE measurements for participants, along with Physical Activity Levels, would allow daily energy requirements to be calculated [13].

Prediction of REE in clinical practice is fundamental to weight management. Elliot et al. [14] found Schofield’s prediction equation based upon body mass as the best predictor of REE in children with DMD (r = 0.6). Alternatively, Shimizu-Fujiwara et al. [4] found both lung function the and body mass to be the best predictor of REE in DMD (r = 0.51, and 0.45 respectively, age range of 10–37 years). It is well established that reduced REE is attributed to loss of muscle mass and FFM with ageing in healthy populations [15]. However, the role of muscles and FFM remains poorly understood in predicting REE in MD. In DMD, psuedohypertrophy presents in older children, which may influence the observed relationship between muscle size and REE. However, recent data has shown lower limb muscles from adults with DMD to be atrophied [16]. Furthermore, data from adults or children with BeMD is limited.

Therefore, the aims of the present investigation were: 1) to compare the REE in adults with and without Becker’s Muscular Dystrophy, 2) to determine whether other physiological parameters correlate with REE in adults with BeMD, for the construction of a more appropriate prediction equation of REE; and 3) To compare current, anthropometric prediction methods of REE with measured REE. The authors hypothesise that: 1) adult males with BeMD will have significantly reduced REE compared to age matched controls; 2) FFM and lower limb muscle ACSA will be the greatest predictors of REE in BeMD; and 3) Current REE predictions equations are unlikely to be suitable for use in a degenerative condition.

## 2. Material and Methods

Twelve healthy adult males (age: 37.8 ±11.9 years, range 21–57 years, height: 1.81 ±0.04 m, Mass: 81.5 ±11.9 Kg, mean ±SD) and 21 men previously diagnosed with BeMD, 9 ambulatory (able to walk with or without an aid) and 12 non-ambulatory (relies on the use of a wheelchair) (age: 39 ±12.7 years range 18–60 years, height: 1.79 ±0.05 m, Mass: 87.2± 16.8 Kg) volunteered to participate in this study. Ethical approval was obtained through the Department of Exercise and Sport Science Ethics Committee, Manchester Metropolitan University, and all participants signed informed consent forms prior to participation. All procedures complied with the World Medical Association Declaration of Helsinki [17].

### 2.1 Procedures

All participants were tested in a single testing session; the control group were tested at Manchester Metropolitan University (Cheshire) and the BeMD group were tested at The Neuromuscular Centre (Winsford, UK). The same equipment was used for both population groups, with the exception of the seated scales for body mass measures in non-ambulatory BeMD participants. All participants were fasted for 12 hours prior to testing, also avoiding caffeine and extensive exercise. All participants were assessed in a seated position to ensure consistency between ambulatory and non-ambulatory participants. Anthropometric and Bioelectrical Impedance measurements were performed first, REE was then measured by indirect calorimetry with a breath-by-breath gas analysis system (Metamax 3B spiroergometer, Cortex, Germany) and analysed using the accompanying software (MetaSoft). Ultrasounds scans were performed post indirect-calorimetry upon the participant’s self-reported dominant leg using a portable ultrasound (MyLab25, Esaote Biomedica, Genoa, Italy). FVC, FEV_1_ and FVC/FEV_1_ ratio measurements were performed last using a Pneumotrac 6800 digital vitalograph and analysed using Spirotrac software (Vitalograph, England). Body fat and fat free mass measurements were completed through Bio-Electrical Impedance using a BodyStat 1500 (BodyStat, England). Both measurement locations were air conditioned, with the ambient temperature set to 20°C.

#### 2.1.1 Anthropometric measurements

All participants height was calculated as point to point (index finger, elbow, shoulder and across midline) span, as to replicate the technique used on non-ambulatory BeMD participants using a 2m tape measure. In order to account for the known discrepancy between standing height and arm span measures, a correction was applied consistent with regression data from adult Caucasian males, the known error of making this correction is 3.5% [18]. Participant height is presented as this corrected value. In the Control group mass was measured by digital scales (Seca model 873, Seca, Germany), BeMD participants were weighed in a digital seated scales system (6875, Detecto, Webb City, Mo, USA). The weight of slings, shoes, splints etc. were subtracted from gross weight post weighing separately.

#### 2.1.2 Body composition measurement

Fat mass and Fat Free Mass were measured using Bioelectrical Impedance (BIA) (Body STAT, 1500) with adhesive electrodes paced on the right hand and foot. Two proximal electrodes were placed between the styloid processes of the right ulna and radius, and between the medial and lateral malleoli of the right ankle. Two Distal electrodes were placed on dorsal surfaces of metacarpals and metatarsals. All participants were measured while seated, and asked to remain still. BIA has previously been used within DMD populations in previous REE studies [10]. BIA has been shown to be valid and reliable in comparison to DEXA, with a Pearson Correlation Coefficient of r = 0.99 in adults of healthy weight, and r = 0.78 in an overweight population [19, 20]. The following equation was used to determine Fat Free Mass:

FFM(kg)=BodyMass(kg)−FatMass(kg)

Equation 1. Fat Free Mass equation: FFM = Fat Free Mass; kg = kilograms.

Body Mass Index (BMI) was calculated using the following equation [21]:

$$BMI\;(kg/m^{2})=Body\;Mass\;(kg)÷Height^{2}(m^{2})$$

Equation 2. BMI Equation: BMI = Body Mass Index; kg = kilograms; m = metres.

#### 2.1.3 Resting energy expenditure

REE was measured by indirect calorimetry using a low dead space, low resistance facemask and volume sensor assembly (MetaMax 3B, Cortex, Leipzig, Germany). Fractional concentrations of O_2_ and CO_2_ were measured by electro-chemical cell, via gas drawn down a capillary line from the facemask. The gas analyser was calibrated according to manufacture guidelines before each test, using calibration gases across the expected range from room air to 16.01% O_2_, and 3.99% CO_2_. Gas volumes were measured through a turbine system attached to the facemask, calibrated using a 3-liter calibration syringe (Hans Rudolph, Inc., Kansas City, USA), and corrected for ambient conditions (pressure and temperature) prior to each test.

**Fig 1:**
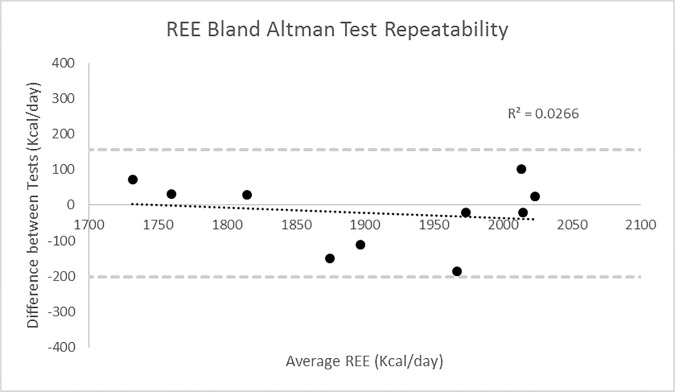
Repeatability of REE in Control Participants (n = 10), Day 1 vs Day 2.

The use of a facemask to calculate REE has been previously validated against the ventilated hood technique (r = 0.91, p<0.001) [22]. In addition, reliability tests of the facemask equipment were performed, with participants measured for 30 minutes while in a fasted state across two separate days at the same time, with the resulting data falling within the Limits of Agreement (LOA) (n = 11. ICC = .87, LOA = +155/-201Kcal) (Fig 1).

**Fig 2:**
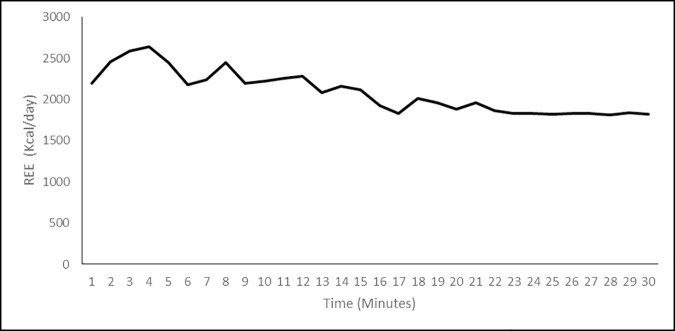
Pilot data of average REE over time in Control Participants.

All participants undertook a 12 hour fast prior to testing. Participants remained with the facemasks attached and seated for up to 30 minutes, with measurements of a plateaued period of breathing taken post 20 minutes. Measurements were taken post 20 minutes because initial wearing of the facemask has been shown to increase breathing rate, while a pilot study showed VO_2_ to plateau post 20 minutes (Fig 2) [23].

The REE values were recorded using the same equation previously used in REE measurements in DMD participants [4], as seen below:

$$REE\;(Kcal/day)=VO_{2}(STPD(l/min))\times 4.825\;(Kcal)\times 60\;(minutes)\times 24\;(hours)$$

Equation 3. REE Measurement Equation: REE = Resting Energy Expenditure; Kcal = Kilocalories; VO_2_ = Oxygen Consumption; STPD = Standard Temperature and Pressure, Dry; l – litres.

Participants were measured post 20 minutes of testing to allow time for Respiratory Quotient (RQ) (ratio of CO_2_ removed from the body to O_2_ consumed by the body) to drop below 0.82. Under conditions where participants RQ is 0.82 or lower, the body consumes 4.825kcal of energy per litre of oxygen [24]. Under normal conditions less than 5% error has been found using the method of indirect calorimetry [25].

Body size has been shown to significantly alter REE of individuals, via an increase in fat free mass. Therefore, to normalise for body size the following equation was also used:

REE(Kcal/day/FFM)=REE(Kcal/day)÷FFM(kg)

Equation 4. REE normalised for FFM: REE = Resting Energy Expenditure; Kcal = Kilocalories; FFM = Fat Free Mass; kg = kilograms.

#### 2.1.4 Ultrasound

Real-time B-Mode ultrasound (MyLab Gamma, Esaote, Cambridgeshire, UK) was used for measurements of muscle length (Lm), Anatomical Cross Sectional Area (ACSA) of the Tibialis Anterior (TA) and Gastrocnemius Medialis (GM) respectively. ACSA was measured using transverse ultrasound scans (width of probe, 7.5-MHz linear array probe) at their largest cross-sectional area, 50% of the GM and 30% of the TA [26]. GM Lm was measured using a tape measure over the skin surface following identification of the visible origin of the GM at the posterior aspect of the femur to the distal formation of the myotendinous junction by use of sagittal pane ultrasonography. TA Lm was measured by the same technique from the lateral condyle of the tibia to the musculotendinous junction.

**Fig 3:**
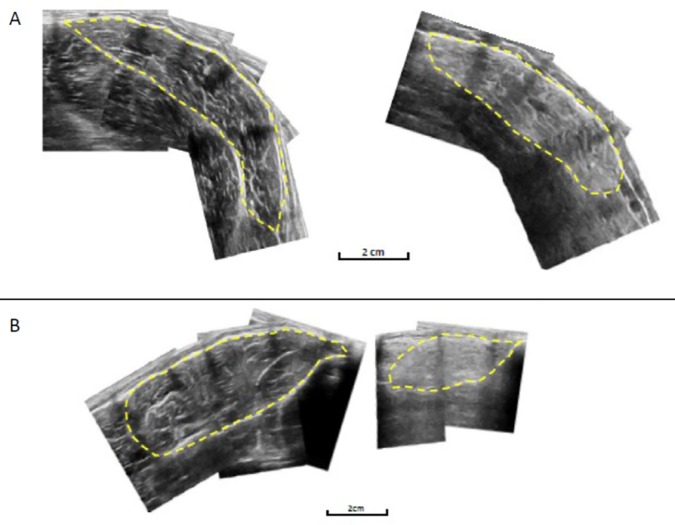
Example of the reconstruction of the GM (A) and TA (B) for Control (Left) and BeMD (Right) participants.

Echoabsoptive tape (Transpore, 3M, USA) was used to project shadows onto the ultrasound image and provide a positional reference. Strips of tape were placed longitudinally across the GM and TA at 50% and 30% of muscle length respectively, at approximately 3cm intervals. A digital recording of the probe moving from the medial to lateral border of the GM and TA was obtained with the probe in a transverse plane. To avoid compression of the muscle, consistent, minimum pressure was applied. The ultrasound was recorded in real time at 25 frames per second (Adobe Premier pro Version 6). At each interval consisting of two reference markers, as shown by shadows from the echoabsoptive tape, individual images were captured using capturing software (Adobe Premier Elements, version 10). The shadows from the echoapsoptive markers allowed the images to be aligned along with the contour of the muscle, and the entire GM and TA ACSA to be recreated in a single image (Fig 3) (Graphic Image Manipulation Program, GIMP Development). The ACSA was then measured using digitising software (ImageJ 1.45, National Institutes of Health, USA). This method of ultrasound to measure ACSA has previously been reported as a valid (0.998) and reliable (0.999) measure in comparison to MRI [26].

#### 2.1.5 Electronic vitalograph

Forced Vital Capacity (FVC) and Forced Expiratory Volume in the first second (FEV_1_) were measured using an electronic spirometer (Pneumotrac, Vitalograph, Bucks, UK). All participants performed the measurements sitting down to replicate the non-ambulatory BeMD participants. Participants had a nose clip attached and the protocol was explained to all participants prior to completion of trials. For each trial participants carried out a maximum inspiratory breath, before breathing out as fast and hard as possible into the mouthpiece. Participants performed three trials and the best performance was accepted. The validity of the Electronic vitalograph is well reported for respiratory function tests in healthy and clinical populations [27].

#### 2.1.6 Resting energy expenditure prediction equations

Two established REE equations were used in comparison to the measured REE values. The simplified Schofield equation [28] using just height and mass, previously identified as the best predictor of REE in children and adolescents with DMD [14]. Alternatively, the Mifflin equation [29] commonly used in healthy individuals was also used. The Mifflin equation includes measures of mass, height and age. Both equations can be seen below:

REE=((17.7×mass(kg)+657)×4.182÷1000)×239

Equation 5. Schofield Equation: REE = Resting Energy Expenditure (Kcal).

REE=10×mass(kg)+6.25×height(cm)−5×age(years)

Equation 6. Mifflin Equation: REE = Resting Energy Expenditure (Kcal)

#### 2.1.7 Physical activity levels

The Bone Specific Physical Activity Questionnaire (BPAQ) is a previously validated, self-administered questionnaire addressing current and previous physical activity levels. The BPAQ algorithms have been previously described and are based upon force loading associated in different physical activity and sports [30]. To eliminate inter-tester variability, BPAQ data was entered and analysed by a single investigator. Activities listed in the BPAQ Calculator were entered, with remaining activities entered as ‘other’ and there perceived impact (low, moderate or high). Current physical activity levels were used for analysis (cBPAQ), which reduced levels have previously been correlated with cardiovascular disease [31]. Although not specifically designed for measurements in individuals with disability, the BPAQ questionnaire has previously been used in Muscular Dystrophy, and shown to correlate significantly with disability specific health questionnaires, such as the Physical Activity Scale for Individuals with Physical Disabilities (PASIPD, [32]). Specifically, within a similar population of adults with BeMD the BPAQ and PASIPD was shown to have significant association (r = 0.71, p = <0.005), and no significant differences between the two physical activity assessment measures (P = 0.404) [33].

#### 2.1.8 Statistical analysis

Data analysis was performed by IBM SPSS Statistics 21 software. The critical level of statistical significance was set at 5%. Tests for parametricity were performed upon all variables. Reliability of REE measured over two days, was calculated using Intraclass Correlation Coefficients, and Bland-Altman analysis (Fig 1, and presented in “Methods” section 2.1.3). Means and standard deviations for all variables can be found in Tables 1, 2 and 3. Independent Student T-Tests were performed between BeMD and Control participants and ANOVA’s performed between BeMD ambulatory status (Ambulatory and non-ambulatory) and control groups, for all variables to find differences. A Two way ANOVA was performed between BeMD age groups (18–40 years vs 41–60 years) for REE. Additionally, Linear and Multiple Regression Analysis was performed to examine the best predictors of REE in BeMD participants. Linear regressions are used to develop possible REE Prediction Equations from measured anthropometric data (FFM, FM etc.). Bland-Altman analysis and explained variance were then used to compare REE measured in the present study using indirect calorimetry, to previously established Prediction Equations, and those developed within the present study.

**Table 1:** Anthropometric measures from adult males with (BeMD) and without (CTRL) Becker’s muscle dystrophy.

	Control	BeMD		
		Combined	Ambulatory	Non-Ambulatory
N	12	21	9	12
Age (Years)	37.8 ±12.0	39.0 ±12.7	39.9 ±13.3	38.4 ±12.2
Height (cm)	179.6 ±5.0	179.2 ±4.0	177.3 ±3.1	177.1 ±3.4
Body Mass (Kg)	80.7±10.2	87.2 ±16.8*	81.0 ±8.6	92.1 ±19.8†
BMI (Kg/m^2^)	25.1 ±3.4	27.5 ±4.9*	25.7 ±2.3	29.3 ±6.0†
Ambulatory (% of participants)	100%	43%*	100%	0%†
Body Fat %	21.0 ±7.6	30.1 ±5.23*	27.4 ±3.6	32.1 ±5.3†
Fat mass (Kg)	17.6 ±8.7	27.1 ±10.6*	22.7 ±5.2	30.5 ±12.5†
FFM (Kg)	63.1 ±3.3	59.5 ±8.6*	55.2 ±7.6	62.2 ±8.3†
BPAQ Score (Current PAL)	5.4 ±3.6	0.6 ±0.8*	1.4 ±0.4	0 ±0†

Data are presented as mean (SD)

*denotes significant difference between groups (P<0.05).

† denotes significant difference from ambulatory BEMD.

BeMD = Becker’s Muscular Dystrophy; BMI = Body Mass Index; BPAQ = Bone Specific Physical Activity Questionnaire; P-P = Point to point; FFM = Fat Free Mass; PAL = Physical Activity Level.

## 3. Results

### 3.1 Anthropometry

BeMD participants were not different in age, height, mass or BMI from CTRL (P>0.05, Table 1). Fat% and fat mass were 43% and 54% greater in BeMD participants compared to CTRL, respectively (P<0.05, Table 1). No significant differences were found between groups for Fat Free Mass. When separated into ambulatory and non-ambulatory groups, significantly greater Body Mass, BMI, Fat%, Fat (Kg) and FFM were found in the non-ambulatory group compared to the ambulatory group (P<0.05, Table 1). BPAQ current physical activity level scores showed BeMD to have 88% lower physical activity than CTRL participants (P<0.05, Table 1). Furthermore, non-ambulatory BeMD participants were significantly less active than ambulatory participants were (P<0.01, Table 1).

The BeMD group had significantly shorter tibia, Ta and GM Length by 7, 15 and 11% repectively (P<0.05, Table 2). TA ACSA was smaller by 43% in BeMD compared to CTRL (P<0.05, Table 2). In contrast, BeMD had a 26% larger GM ACSA compared to CTRL (P<0.05, Table 2).

**Table 2:** Lower limb muscle size measures from adult males with (BeMD) and without (Control) Becker’s muscle dystrophy.

	Control	BeMD		
		Combined	Ambulatory	Non-Ambulatory
Tibia Length (cm)	44.0 ±1.3	40.8 ±3.0*	41.7 ±2.9	40.2 ±2.8
TA Length (cm)	25.8 ±1.9	21.7 ±3.1*	22.6 ±2.9	21.0 ±3.2
TA ACSA (cm^2^)	9.9 ±1.9	5.7 ±1.5*	5.6 ±1.2	5.7 ±1.8
GM Length (cm)	24.0 ±2.2	21.3 ±3.8*	21.5 ±2.4	21.2 ±4.7
GM ACSA (cm^2^)	15.4 ±2.9	19.3 ±9.7*	15.5 ±4.3	22.1 ±11.7†

Data are presented as mean (SD)

*denotes significant difference from control groups (P<0.05).

† denotes significant difference from ambulatory BEMD. TA–Tibialis Anterior; ACSA–Anatomical Cross Sectional Area; GM–Gastrocnemius Medialis.

No difference was found between ambulatory and non-ambulatory BeMD participants for Tibia Length, TA ACSA, TA length or GM Length (Table 2). Non-ambulatory BeMD participants showed 30% larger GM ACSA compared to ambulatory BeMD (P<0.05, Table 2).

### 3.2 Respiratory measures

FVC and FEV_1_ were 28% and 27% lower for BeMD participants compared to controls, respectively (P<0.05, Table 3). No differences were found between groups for FVC/FEV_1_ (Table 3). 36% and 37% smaller FVC and FEV_1_ values were observed for non-ambulatory BeMD compared to ambulatory BeMD (p<0.05, Table 3), however no differences were found in FVC/FEV_1_ (p>0.05).

**Table 3:** Respiratory function, and resting calorimeter measures from adult males with (BeMD) and without (Control) Becker’s muscle dystrophy.

	Control	BeMD		
		Combined	Ambulatory	Non-Ambulatory
FEV_1_ (L)	4.2 ± 0.7	3.1 ± 1.2*	3.6 ± 0.6	2.7 ± 1.4
FVC (L)	5.1 ± 0.4	3.6 ± 1.4*	4.3 ± 0.8	3.2 ± 1.5†
FEV_1_/FVC (%)	83.2 ± 9.7	85.3 ± 8.8	85.1 ± 5.3	85.3 ± 10.7
REE (Kcal/Day)	1913.6 ± 203.2	1786 ± 324.1	1676 ± 246.3	1869 ±352.2
REE (Kcal/Day/FFM)	30.0 ± 2.3	30.0 ± 2.1	30.1 ± 2.2	29.9 ± 2.1

FEV_1_ –Forced Expiratory Volume in 1^st^ Second; FVC–Forced Vital Capacity; REE–Resting Energy Expenditure; Kcal–Kilocalories; FFM–Fat Free Mass. Data are presented as mean (SD)

*denotes significant difference between groups (P<0.05).

### 3.3 Energy expenditure

No significant differences were found between BeMD or CTRL groups for REE or REE/FFM (p>0.05, Table 3). Within BeMD participants, there was no difference between age groups (18–40 and 41–60 years old) for REE, 1885 Kcal/Day and 1853 Kcal/Day, REE/FFM, 29.22 Kcal/FFM/Day and 30.78 Kcal/FFM/Day, or ambulatory status (Table 4).

**Table 4:** Prediction Equations correlation with measured REE in Adults with BeMD.

Equation	REE (Kcal/Day)	Bias (Kcal)	Limits of Agreement (-/+(Kcal)	Correlation (R^2^)
Measured	1786 (324)	n/a	n/a	n/a
Schofield et al (1985)	2514 (340)*	-728	-1126, -328	0.66
Mifflin et al (1990)	1801 (197)	-8	-429, 412	0.58
FFM x 34.572–270.51	1787 (299)	0	-241, 241	0.86
BM x 15.652 + 421.15	1786 (279)	0.02	-362, 362	0.66

Values shown are measured and predicted REE and correlation values (R^2^).

*Denotes significant difference between measured and estimated REE.

REE = Resting Energy Expenditure; Kcal = Kilocalories; FFM = Fat Free Mass; BM = Body Mass.

**Fig 4:**
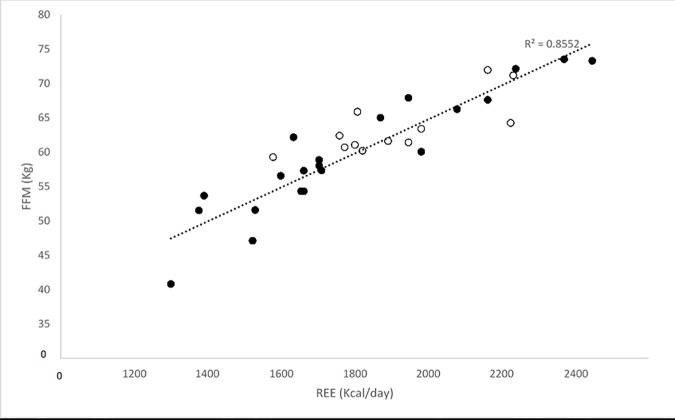
REE and FFM relationship in BeMD (closed circles) and Control participants (open circles). FFM = Fat Free Mass; Kg = Kilograms; REE = Resting Energy Expenditure; Kcal = Kilocalories.

**Fig 5:**
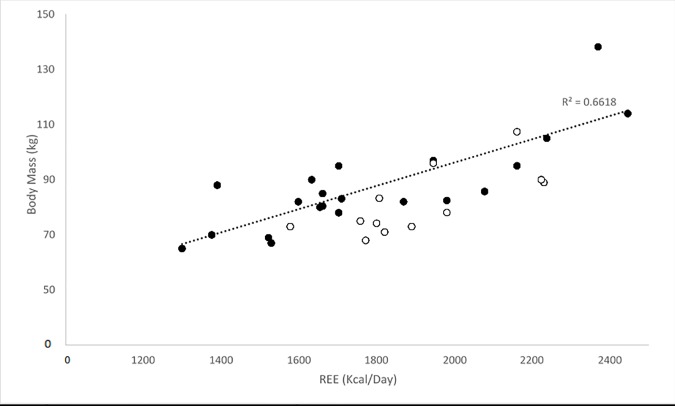
REE and Body Mass (Kg) relationship in BeMD (closed circles) and Control (open circles) participants. Kg = Kilograms; REE = Resting Energy Expenditure; Kcal = Kilocalories.

**Fig 6:**
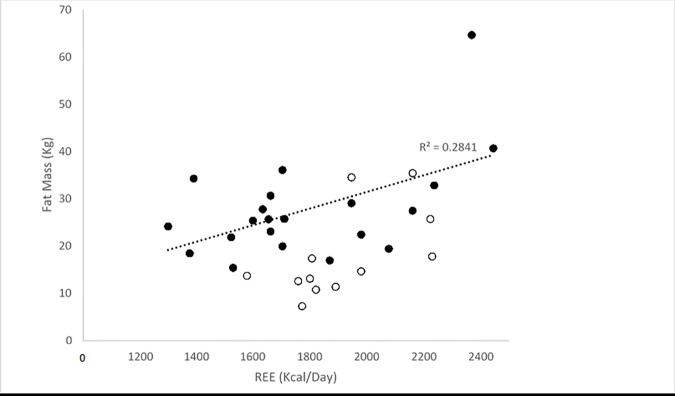
REE and Fat Mass (Kg) relationship in BeMD (closed circles) and Control (open circles) participants. Kg = Kilograms; REE = Resting Energy Expenditure; Kcal = Kilocalories.

**Fig 7:**
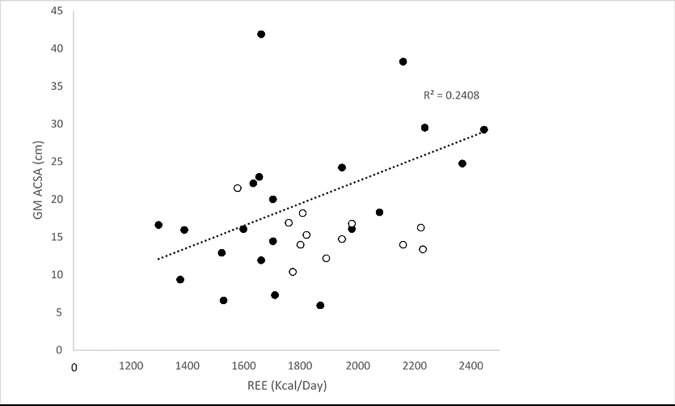
Relationship between GM ACSA and REE in BeMD (closed circles) and Control (open circles) participants. GM = Gastrocnemius Medialis; ACSA = Anatomical Cross Sectional Area; cm = centimetres; REE = Resting Energy Expenditure; Kcal = Kilocalories.

Within the BeMD participant’s, linear regressions revealed significant relationships between four variables and REE (Kcal/day). The strongest correlation was found between FFM and REE (Kcal/day) (R^2^ = 0.85; P<0.05; Fig 4). Similarly, there were positive correlations between body mass (R^2^ = 0.66; P<0.05; Fig 5), Fat mass (R^2^ = 0.28; P<0.05; Fig 6) and GM ACSA (R^2^ = 0.24; P<0.05; Fig 7) with REE (Kcal/day). Compared to FFM (the strongest predictor of REE) multiple regression showed no further benefit of including all anthropometric variables (R^2^ = 0.87).

Within BeMD participants existing REE prediction equations showed that REE estimated from mass (the Schofield equation) to be 41% more than the measured REE values (p<0.05; Table 4). REE estimated from Mass, height and age (the Mifflin equation) was not significantly different from the measured REE values (p>0.05; Table 4). Based on the present participants, we found FFM to be the best predictor of REE (Equation 7: REE = 34.57 x FFM—270.5), showing no difference from measured values. Body mass estimates of REE within the present BeMD participants (Equation 8: REE = Body Mass x 15.652 + 421.15) were also not significantly different from measured REE, however not to the extent of the FFM equation.

REE=FFM×34.57−270.51

Equation 7. FFM Based Prediction Equation of REE in BeMD Adults. REE = Resting Energy Expenditure (Kcal); FFM = Fat Free Mass (Kg).

REE=BM×15.652+421.15

Equation 8. Body Mass Based Prediction Equation of REE in BeMD Adults. REE = Resting Energy Expenditure (Kcal); BM = Body Mass (Kg).

### 3.4 Co-variables

Of the co-variables measured, equations involving body mass and FFM were found to show strong correlations with measured REE (Body Mass p>0.05; r = 0.81; FFM p>0.05; r = 0.91; See Table 4). The Mifflin predicted REE was 1801 Kcal/Day±202 and was not significantly different from the measured REE 1786 Kcal/Day±324. In contrast, the Schofield prediction equation, 2514 Kcal/Day±340, was significantly greater than the measured REE. The REE prediction equations produced Pearson’s Correlation Coefficients of 0.81 and 0.79 for the Schofield and Mifflin Equations, respectively. Bland-Altman analysis illustrated the systematic bias and random error, between the prediction equations and measured REE (Table 4). There was a 40% greater REE when the Schofield prediction equation was used compared to the measured REE in BeMD (P<0.05), indicating substantial systematic bias. In contrast, the REE estimated from the Mifflin prediction equation showed no difference from the measured REE (P>0.05).

## 4. Discussion

The present study had three main findings, 1) REE was not different between adult males with and without BeMD; 2) FFM showed the closest association to REE in the BeMD participants; 3) Of the two previously used REE prediction equations, one (using body mass, height and age) was valid within adult males with BeMD. However (consistent with finding 2) from the present data, prediction equations based upon FFM were found to be the best predictor of REE in BeMD. We also present here novel anthropometric and muscle data from adult males with BeMD, which is somewhat consistent with previous observations from the muscles of children with MD.

The requirement for nutritional guidelines in MD is particularly relevant given the evidenced increased fat mass of those with the condition in the present study and previous [16, 34]. This is likely a result of calorific imbalance [35] and reduced physical activity levels observed here in adults with BeMD and previously in adults with DMD [36]. Weight gain is common in adolescents with MD, and often exacerbated by the use of corticosteroid treatment, such as deflazacort and prednisone [37, 38]; however, no participants in the current study have any history of corticosteroid treatment. Lower body mass has been previously reported in adults with DMD [16], however this was attributed to the loss of FFM associated with the condition. In contrast, the current adult BeMD participants presented with a greater body mass than controls, which is likely to be a combination of maintained FFM, as well as increased Fat Mass compared to the controls. The higher fat mass in the present BeMD group is likely a combination of reduced physical activity (discussed below) and excessive calorific intake (although calorific intake was not measured in the present study). Based on the present observations, calorific guidelines using FFM may help to reduce any negative impact on cardiac function [35] and muscle degradation [37] associated with excessive body fat.

In the present study, within BeMD there was a strong correlation between FFM and REE, however REE did not differ between any groups. Additionally, no differences were seen between groups, when REE was normalised for FFM. Consistent with this, the largest correlations between anthropometric measures and REE were found with FFM and BM. Similar to the non-dystrophic control population, the present study showed that individuals with BeMD had a daily resting calorific requirement of 1786 Kcal/day. This is in contrast to previous data from adults with DMD who have been shown to have significantly reduced REE with age [4, 11]. For example, Shimizu-Fujiwara et al. [4] and Gonzalez-Bermejo et al. [11], both reported REE to be significantly lower than control groups with adult DMD patients presenting with 970–1239 and 1089 Kcal/day [4, 11], respectively. The higher REE in the present study compared to those in the DMD population, is likely a reflection of the less severe nature of BeMD compared to DMD and their higher FFM compared to DMD. Calorific guidelines for those with BeMD should therefore, not be based on previous studies in DMD. Where previous studies have used body mass to estimate REE in MD, REE was predicted to be 2530Kcal/Day using body mass [14]. These are significantly higher than those measured in the current study (1786±324 kcal/day) and not only highlights the requirement for nutritional guidelines specific to BeMD, but is consistent with the lower validity of using body mass estimates of REE over FFM in the present study.

Previous DMD studies [4] have used the Schofield prediction equation to estimate REE in adults, while they also showed FEV_1_ to be a strong physiological predictor of REE. However, within the current study the Mifflin equation (commonly used in non-dystrophic populations) was found to be a more appropriate method of REE prediction in adults with BeMD, and measures of FFM and BM were shown to be much better physiological predictors of REE then FEV_1_. The Schofield [28] prediction method did overestimate REE in BeMD, which may have implications for daily calorific intakes and long-term health in BeMD. Additively, the wider confidence intervals of the Mifflin equation and BM equation suggest where possible, FFM is adopted in future methods for predicting REE in adults with BeMD.

Our findings of a lower FFM in BeMD compared to previous is consistent with previous measures of lower muscle, and bone mass in BeMD compared to controls [33]. What was somewhat surprising was the fact that within BeMD, Non-ambulatory participants had higher FFM than ambulatory BeMD participants. Non-ambulatory participants would be expected to have reduced FFM (and therefore REE) due to lower physical activity levels [39]. The greater FFM and GM ACSA in the present non-ambulatory BeMD participants may represent psuedohypertrophy, an inflammatory response from increased muscle degeneration previously reported in adolescents with DMD [40]. It is possible, given the similar loci of impairments within the dystrophin protein [1], that the more severe forms of BeMD, specifically the non-ambulatory participants in the present study, may present with pseudohypertrophy similar to adolescents with DMD.

Despite the well-established symptoms and progression of the condition [34, 41], there is at present very few studies comparing neuromuscular and respiratory measures between adult males with and without BeMD. Our respiratory data (FEV_1_, FVC, and FEV_1_/FVC) is consistent with others who have reported relative impairments in adults with MD [42]. We have observed significant impairments in FEV_1_ and FVC in BeMD compared to controls. Respiratory function, and its impact on sleep apnoea (an obstructive condition amplified by excessive weight) and nocturnal hypoventilation (airway restriction associated with muscle fatigue against abdominal pressure when supine), has long been recognised in DMD, and highly associated with quality of life and life expectancy [43, 44]. Future studies, particularly intervention studies, should focus on respiratory impairment and their responses in adults with BeMD.

Measures of TA ACSA in the present study show that BeMD were smaller than control participants are similar to those reported previously [33], and aligns itself with the classical muscle wasting definition of the condition; GM ACSA has been commented upon previously, and is heavily influenced by the non-ambulatory nature of this sample [16]. It is possible that similar to adults with DMD [16], the progression of BeMD may show atrophy within the GM, however these are not presented in the current adults who are all aged < 55 years. More longitudinal studies are required, tracking changes in muscle, FFM and BM to further understand the condition.

Within the present study there were two primary limitations, 1) the use of BIA, and 2) no measure of calorific intake. BIA remains unvalidated in BeMD, however it has been shown previously to be a strong predictor of FFM in children with DMD and was suggested as a tool to measure changes in nutritional status [45]. We recently addressed similar concerns when measuring bone health [33] and noted that contractures, joint stiffness and lower mobility in general make more stringent measures of FFM, such as DEXA, difficult to obtain in MD. However, in terms of guidelines for calorific intake, we also feel that BIA is a more available and cost effective tool than the alternative [46]. In addition, calorific intake and calorific energy expenditure, through physical activity, were not measured in the current study. Although this does not impact on our main findings in terms of REE, knowledge of calorific intake and expenditure would allow us to make stronger conclusions regarding the mechanisms and determinants of higher fat mass in BeMD compared to controls in the present study.

The main clinical implication from our present study is that fat mass and BMI are higher in BeMD than controls, and prediction equations based on anthropometric measures may be useful in addressing this higher fat mass. Evident from our anthropometric data, we have observed that fat mass, BMI and relative fat mass is higher in adults with BeMD, particularly in non-ambulatory participants. Combined with our finding that there is no difference in REE from controls, greater dietary considerations and advice are needed for adults with BeMD in order to control for weight gain, and any subsequent co-morbidities associated with this accrued fat mass. Specific to the present data, our prediction equation using FFM may be useful in addressing some of the issues associated with higher body fat and fat mass observed in the present BeMD participants; however longitudinal data on weight management and dietary requirements in BeMD is required.

In conclusion, while no direct differences were found in measured REE between adults with and without BeMD, the increased levels of fat mass in the BeMD group, and large differences found in previously established REE prediction equations highlight the requirement for specific nutritional guidelines within BeMD adults. The strong correlation found between measured REE and FFM means FFM should become the basis for prediction REE equations in BeMD adults. Furthermore, physical activity should be encouraged, where possible, in an attempt to maintain FFM and reduce calorific imbalances, as well as to prevent possible cardiovascular and respiratory health implications.

## Supporting Information

S1 File Basic Data. (XLSX)

## Abbreviations

REE: Resting Energy Expenditure
MD: Muscular Dystrophy
BeMD: Becker’s Muscular Dystrophy
DMD: Duchenne Muscular Dystrophy
FFM: Fat Free Mass
ACSA: Anatomical Cross Sectional Area
Lm: Muscle Length
TA: Tibialis Anterior
GM: Gastrocnemius Medialis
FVC: Forced Vital Capacity
FEV_1_: Forced Expiratory Volume 1 second

## References

[1] Koenig M, Beggs AH, Moyer M, Scherpf S, Heindrich K, Bettecken T, et al The molecular basis for Duchenne versus Becker muscular dystrophy: correlation of severity with type of deletion. American journal of human genetics. 1989;45(4):498 2491009 PMC1683519

[2] Hoffman EP, Brown RH, Kunkel LM. Dystrophin: the protein product of the Duchenne muscular dystrophy locus. Cell. 1987;51(6):919–28. 3319190 10.1016/0092-8674(87)90579-4

[3] Emery AEH. Population frequencies of inherited neuromuscular diseases—a world survey. Neuromuscular disorders. 1991;1(1):19–29. 1822774 10.1016/0960-8966(91)90039-u

[4] Shimizu-Fujiwara M, Komaki H, Nakagawa E, Mori-Yoshimura M, Oya Y, Fujisaki T, et al Decreased resting energy expenditure in patients with Duchenne muscular dystrophy. Brain and Development. 2012;34(3):206–12. 10.1016/j.braindev.2011.05.005 21632191

[5] Miles JM, editor Energy expenditure in hospitalized patients: implications for nutritional support 2006: Elsevier. 10.4065/81.6.809 16770981

[6] Marra M, Montagnese C, Sammarco R, Amato V, Della Valle E, Franzese A, et al Accuracy of Predictive Equations for Estimating Resting Energy Expenditure in Obese Adolescents. The Journal of pediatrics. 2015;166(6):1390–6. 10.1016/j.jpeds.2015.03.013 25872963

[7] Astrup A, Buemann B, Christensen NJ, Madsen J, Gluud C, Bennett P, et al The contribution of body composition, substrates, and hormones to the variability in energy expenditure and substrate utilization in premenopausal women. The Journal of Clinical Endocrinology & Metabolism. 1992;74(2):279–86. 1530952 10.1210/jcem.74.2.1530952

[8] Garby L, Lammert O. Between-subjects variation in energy expenditure: estimation of the effect of variation in organ size. European journal of clinical nutrition. 1994;48(5):376–8. 8055854

[9] Heymsfield SB, Gallagher D, Kotler DP, Wang Z, Allison DB, Heshka S. Body-size dependence of resting energy expenditure can be attributed to nonenergetic homogeneity of fat-free mass. American Journal of Physiology-Endocrinology And Metabolism. 2002;282(1):E132–E8. 11739093 10.1152/ajpendo.2002.282.1.E132

[10] Wren TAL, Bluml S, Tseng-Ong L, Gilsanz V. Three-point technique of fat quantification of muscle tissue as a marker of disease progression in Duchenne muscular dystrophy: preliminary study. American Journal of Roentgenology. 2008;190(1):W8–W12. 10.2214/AJR.07.2732 18094282

[11] Gonzalez-Bermejo J, Lofaso F, Falaize L, Lejaille M, Raphael JC, Similowski T, et al Resting energy expenditure in Duchenne patients using home mechanical ventilation. European Respiratory Journal. 2005;25(4):682–7. 10.1183/09031936.05.00031304 15802343

[12] Evans WJ. Skeletal muscle loss: cachexia, sarcopenia, and inactivity. The American journal of clinical nutrition. 2010;91(4):1123S–7S. 10.3945/ajcn.2010.28608A 20164314

[13] World Health O. Human energy requirements: NS; 2005.

[14] Elliott SA, Davidson ZE, Davies PSW, Truby H. Predicting resting energy expenditure in boys with Duchenne muscular dystrophy. european journal of paediatric neurology. 2012;16(6):631–5. 10.1016/j.ejpn.2012.02.011 22497714

[15] Bosy-Westphal A, Eichhorn C, Kutzner D, Illner K, Heller M, Müller MJ. The age-related decline in resting energy expenditure in humans is due to the loss of fat-free mass and to alterations in its metabolically active components. The Journal of nutrition. 2003;133(7):2356–62. 12840206 10.1093/jn/133.7.2356

[16] Morse CI, Smith J, Denny A, Tweedale J, Searle ND. Gastrocnemius medialis muscle architecture and physiological cross sectional area in adult males with Duchenne muscular dystrophy. J Musculoskelet Neuronal Interact. 2015;15(2):154–60. 26032207 PMC5133718

[17] World Medical A. World Medical Association Declaration of Helsinki: ethical principles for medical research involving human subjects. Jama. 2013;310(20):2191 10.1001/jama.2013.281053 24141714

[18] Reeves S, Varakamin C, Henry C. The relationship between arm-span measurement and height with special reference to gender and ethnicity. European Journal of Clinical Nutrition. 1996;50(6):398–400. 8793422

[19] Okasora K, Takaya R, Tokuda M, Fukunaga Y, Oguni T, Tanaka H, et al Comparison of bioelectrical impedance analysis and dual energy X‐ray absorptiometry for assessment of body composition in children. Pediatrics international. 1999;41(2):121–5. 10221012 10.1046/j.1442-200x.1999.4121048.x

[20] Sun G, French CR, Martin GR, Younghusband B, Green RC, Xie Y-g, et al Comparison of multifrequency bioelectrical impedance analysis with dual-energy X-ray absorptiometry for assessment of percentage body fat in a large, healthy population. The American journal of clinical nutrition. 2005;81(1):74–8. 15640463 10.1093/ajcn/81.1.74

[21] McCabe MP, Ricciardelli LA, Parent P. BODY MASS INDEX. Eating Disorders: An Encyclopedia of Causes, Treatment, and Prevention. 2013;34:90.

[22] McAnena OJ, Harvey LP, Katzeff HL, Daly JM. Indirect calorimetry: comparison of hood and mask systems for measuring resting energy expenditure in healthy volunteers. Journal of Parenteral and Enteral Nutrition. 1986;10(6):555–7. 3795448 10.1177/0148607186010006555

[23] Isbell TR, Klesges RC, Meyers AW, Klesges LM. Measurement reliability and reactivity using repeated measurements of resting energy expenditure with a face mask, mouthpiece, and ventilated canopy. Journal of Parenteral and Enteral Nutrition. 1991;15(2):165–8. 2051556 10.1177/0148607191015002165

[24] Igawa S, Sakamaki M, Miyazaki M. Examination of the reliability of the portable calorimeter. Clinical and Experimental Pharmacology and Physiology. 2002;29(S4):S13–S5. 29537678 10.1046/j.1440-1681.29.s4.7.x

[25] Tamura T, Ichinoseki N, Yoshimura T, Torii Y. Development and evaluation of a simple calorimeter for the measurement of resting metabolism. Clinical and Experimental Pharmacology and Physiology. 2002;29(S4):S2–S6. 29537677 10.1046/j.1440-1681.29.s4.3.x

[26] Reeves ND, Maganaris CN, Narici MV. Ultrasonographic assessment of human skeletal muscle size. European journal of applied physiology. 2004;91(1):116–8. 10.1007/s00421-003-0961-9 14639480

[27] Fonseca JA, Costa-Pereira A, Delgado L, Silva LN, Magalhaes M, Castel-Branco MG, et al Pulmonary function electronic monitoring devices: a randomized agreement study. CHEST Journal. 2005;128(3):1258–65. 10.1378/chest.128.3.1258 16162716

[28] Schofield WN. Predicting basal metabolic rate, new standards and review of previous work. Human nutrition Clinical nutrition. 1984;39:5–41. 4044297

[29] Mifflin MD, St Jeor ST, Hill LA, Scott BJ, Daugherty SA, Koh YO. A new predictive equation for resting energy expenditure in healthy individuals. The American journal of clinical nutrition. 1990;51(2):241–7. 2305711 10.1093/ajcn/51.2.241

[30] Weeks BK, Beck BR. The BPAQ: a bone-specific physical activity assessment instrument. Osteoporosis International. 2008;19(11):1567–77. 10.1007/s00198-008-0606-2 18414964

[31] Weeks BK, Purvis M, Beck BR. Physical activity estimated by the bone-specific physical activity questionnaire is also associated with cardiovascular risk. European journal of sport science. 2016:1–8. 10.1080/17461391.2016.1153726 26937743

[32] Washburn RA, Zhu W, McAuley E, Frogley M, Figoni SF. The physical activity scale for individuals with physical disabilities: development and evaluation. Archives of physical medicine and rehabilitation. 2002;83(2):193–200. 11833022 10.1053/apmr.2002.27467

[33] Morse CI, Smith J., Dennym A., Tweedale J., Searle N.D., Winwood K., Onambele-Pearson G.L. Bone health measured using quantitative ultrasonography in adult males with muscular dystrophy. Journal of Musculoskeletal and Neuronal Interactions. 2016;16(4):339–47. 27973386 PMC5259575

[34] Huml RA. Muscular Dystrophy: A Concise Guide: Springer; 2015.

[35] Goldstein M, Meyer S, Freund HR. Effects of overfeeding in children with muscle dystrophies. Journal of Parenteral and Enteral Nutrition. 1989;13(6):603–7. 2515307 10.1177/0148607189013006603

[36] McDonald CM, Widman LM, Walsh DD, Walsh SA, Abresch RT. Use of step activity monitoring for continuous physical activity assessment in boys with Duchenne muscular dystrophy. Archives of physical medicine and rehabilitation. 2005;86(4):802–8. 10.1016/j.apmr.2004.10.012 15827935

[37] Fenichel GM, Florence JM, Pestronk A, Mendell JR, Moxley RT, Griggs RC, et al Long‐term benefit from prednisone therapy in Duchenne muscular dystrophy. Neurology. 1991;41(12):1874–. 1745340 10.1212/wnl.41.12.1874

[38] Bonifati MD, Ruzza G, Bonometto P, Berardinelli A, Gorni K, Orcesi S, et al A multicenter, double‐blind, randomized trial of deflazacort versus prednisone in Duchenne muscular dystrophy. Muscle & nerve. 2000;23(9):1344–7. 10951436 10.1002/1097-4598(200009)23:9<1344::aid-mus4>3.0.co;2-f

[39] Hachisuka K, Umezu Y, Ogata H. Disuse muscle atrophy of lower limbs in hemiplegic patients. Archives of physical medicine and rehabilitation. 1997;78(1):13–8. 9014951 10.1016/s0003-9993(97)90003-4

[40] Jones DA, Round JM, Edwards RHT, Grindwood SR, Tofts PS. Size and composition of the calf and quadriceps muscles in Duchenne muscular dystrophy: a tomographic and histochemical study. Journal of the neurological sciences. 1983;60(2):307–22. 6886735 10.1016/0022-510x(83)90071-0

[41] McDonald CM, Abresch RT, Carter GT, Fowler WM Jr, Johnson ER, Kilmer DD. Profiles of neuromuscular diseases: Becker's muscular dystrophy. American journal of physical medicine & rehabilitation. 1995;74(5):S104. 7576425 10.1097/00002060-199509001-00004

[42] Hapke EJ, Meek JC, Jacobs J. Pulmonary function in progressive muscular dystrophy. Chest. 1972;61(1):41–7. Epub 1972/01/01. 5049500 10.1378/chest.61.1.41

[43] Jeronimo G, Nozoe KT, Polesel DN, Moreira GA, Tufik S, Andersen ML. Impact of corticotherapy, nutrition, and sleep disorder on quality of life of patients with Duchenne muscular dystrophy. Nutrition. 2016;32(3):391–3. 10.1016/j.nut.2015.09.004 26701140

[44] Eagle M, Baudouin SV, Chandler C, Giddings DR, Bullock R, Bushby K. Survival in Duchenne muscular dystrophy: improvements in life expectancy since 1967 and the impact of home nocturnal ventilation. Neuromuscular disorders. 2002;12(10):926–9. 12467747 10.1016/s0960-8966(02)00140-2

[45] Mok E, Letellier G, Cuisset J-M, Denjean A, Gottrand F, Hankard R. Assessing change in body composition in children with Duchenne muscular dystrophy: anthropometry and bioelectrical impedance analysis versus dual-energy X-ray absorptiometry. Clinical nutrition. 2010;29(5):633–8. 10.1016/j.clnu.2010.03.011 20427103

[46] McDonald CM, Carter GT, Abresch RT, Widman L, Styne DM, Warden N, et al Body composition and water compartment measurements in boys with Duchenne muscular dystrophy. American journal of physical medicine & rehabilitation. 2005;84(7):483–91. 15973084 10.1097/01.phm.0000166880.91117.04

